# Large Intestine Histopathology of Pegylated-Interferon-Alpha Plus Ribavirin Treated Chronic Hepatitis C Patients

**DOI:** 10.1155/2008/637517

**Published:** 2008-03-19

**Authors:** Konstantinos D. Pantazis, Ioannis S. Elefsiniotis, Dimitrios Papaioannou, Hero Brokalaki, Gerasimos Bonatsos, Christos Mavrogiannis

**Affiliations:** ^1^Department of Internal Medicine and Hepatogastroenterology, Faculty of Nursing, Helena Venizelou Hospital, University of Athens, 11521 Athens, Greece; ^2^Department of Pathology, Hygeia Hospital, 15123 Athens, Greece

Gastrointestinal disorders (especially diarrhea) are observed in a proportion of patients treated with the currently approved combination treatment for chronic hepatitis C (CHC), pegylated-interferon alpha (PEG-IFNa) plus ribavirin (RIB) [1]. Several reports suggest that the presence of inflammatory bowel disease (IBD) is not a contraindication for interferon-alpha- (IFNa-) based treatments [2, 3]. Furthermore, a randomised placebo-controlled trial of PEG-IFNa in patients with active ulcerative colitis concluded that PEG-IFNa is safe but not effective treatment for these patients [4]. On the contrary, several reports [5, 6, 7, 8] revealed that treatment of chronic viral hepatitis with IFNa or PEG-IFNa with or without RIB was related with the onset of clinically and histologically confirmed acute colitis of the IBD type. To our knowledge the effect of chronic hepatitis C virus (HCV) infection or PEG-IFNa plus RIB combination treatment on large intestine histopathology has not been investigated, within a clinical trial. The principal aim of our study was to investigate the effect of PEG-IFNa plus RIB treatment on the large intestine histology of treated CHC patients.

Twenty-four treatment-naïve CHC patients with serologically (antiHCV-positive, Abbott Laboratories, Abbott Park, Ill, USA), virologically (serum HCV-RNA detection, Cobas Amplicor HCV test, version 2, Roche Diagnostics, Branchburg, NJ, USA), and histologically (liver biopsy-Ishak scoring system) confirmed CHC and no contraindication of receiving combination treatment with PEG-IFNα2b and RIB were enrolled in this pilot study. All patients were treated with weight-based dosing of pegylated interferon-a2b (Peg-Intron, 1.5*μ*g/kg/week) and genotype-related ribavirin dose (Rebetol, 800 mg/day for genotype 2/3 and 1000–1200 mg/day for genotype 1/4-infected patients, depending on baseline body weight < or ≥ 85 kg, resp.) for 24 weeks. Patients were evaluated for the presence of gastrointestinal symptoms, by receiving a detailed history, before the beginning of treatment, during the treatment period and at week 24 of treatment. The study population underwent recto-sigmoidoscopic examination prior to the beginning of the treatment schedule. Three to five biopsy specimens were taken from the rectosigmoid area.

Histological findings characteristic of inflammation as well as the presence of architectural disorders and the quantity of mucus production, the presence of ulcerations and Paneth cells as well as the numbers of lymphoid follicles in every biopsy specimen were evaluated. Fifteen patients underwent the same procedure after the completion of 24-week treatment course and three of them were also evaluated during treatment because of diarrhea. Ten age-, sex-, and BMI-matched healthy subjects (control group) underwent recto-sigmoidoscopic examination and biopsy. All controls had no history of inflammatory bowel disease or other gastrointestinal diseases, did not receive any medication and did not report symptoms from the gastrointestinal tract. Written informed consent was obtained from each participant for his or her participation in the study. The study conformed to the ethical guidelines of the 1975 Declaration of Helsinki.

No pathological macroscopic (endoscopic) findings were identified at baseline as well as in endoscopic re-evaluation in either patients or controls. CHC patients had no statistically significant difference in the presence of bowel inflammatory infiltration compared to controls as shown in Table 1. It is important to note that a *respectable proportion* of CHC patients exhibited architectural disorder and *decreased* mucus production (25%) as well as presence of Paneth cells (16.7%), compared to controls, but this does not reach statistical significance possibly due to the small sample size of the study population. This finding needs further investigation because of the documental lymphotropism of HCV and the well-known HCV-related autoimmune manifestations.

No patient reported diarrhea before the initiation of treatment whereas 3 patients reported diarrhea during the treatment course. In particular, the first patient reported diarrhea at week 6 of treatment, the second one at week 12, and the third one at week 14. Baseline histology was normal in all three patients. Histopathological evaluation of the large intestine at the onset of symptoms revealed mild chronic nonspecific colitis in the first patient (Figure 1) and normal large intestine histology in the other two patients, respectively. The same findings were repeated in these patients in the histological re-evaluation at week 24 of treatment. Diarrhea spontaneously resolved in all of them within 5–7 days and none of them followed treatment discontinuation.

There was no statistically significant difference between the percentage of patients with large intestine inflammation before the initiation and at week 24 of treatment (66.7% versus 53.3% resp., *P* = .71). Particularly, 6/15 patients exhibited mild inflammatory infiltration before the initiation as well as at week 24 and 3/15 exhibited absence of inflammation both before and at week 24 of treatment. Moreover, 4/15 positive patients for the presence of inflammation before treatment had normal large intestine histology at week 24 and finally, 2/15 patients had no findings of inflammation before treatment but exhibited mild nonspecific inflammatory lesions at week 24. No statistically significant differences were observed before the beginning and at week 24 of combination treatment regarding architectural disorder and decrease of mucus production (*P* = .70), presence of Paneth cells (*P* = .10), and the number of lymphoid follicles in every biopsy specimen (*P* = .97) as shown in Table 2. Interestingly none of 4 patients with detectable Paneth cell at baseline evaluation exhibits presence of them at week 24. This finding needs further investigation in large-scale studies because of the proposed importance of these multifaceted cells in the pathophysiology of IBD [9].

In conclusion, according to the preliminary results of our pilot study, it seems that the immunomodulatory-antiviral treatment of CHC with PEG-IFNa2b plus ribavirin for 24 weeks possibly does not significantly affect the large intestine histology of treated patients, despite the appearance of symptoms from the gastrointestinal tract in a subgroup of them. The effect of chronic HCV infection in the intestine histopathology needs further investigation.

## Figures and Tables

**Figure 1:**
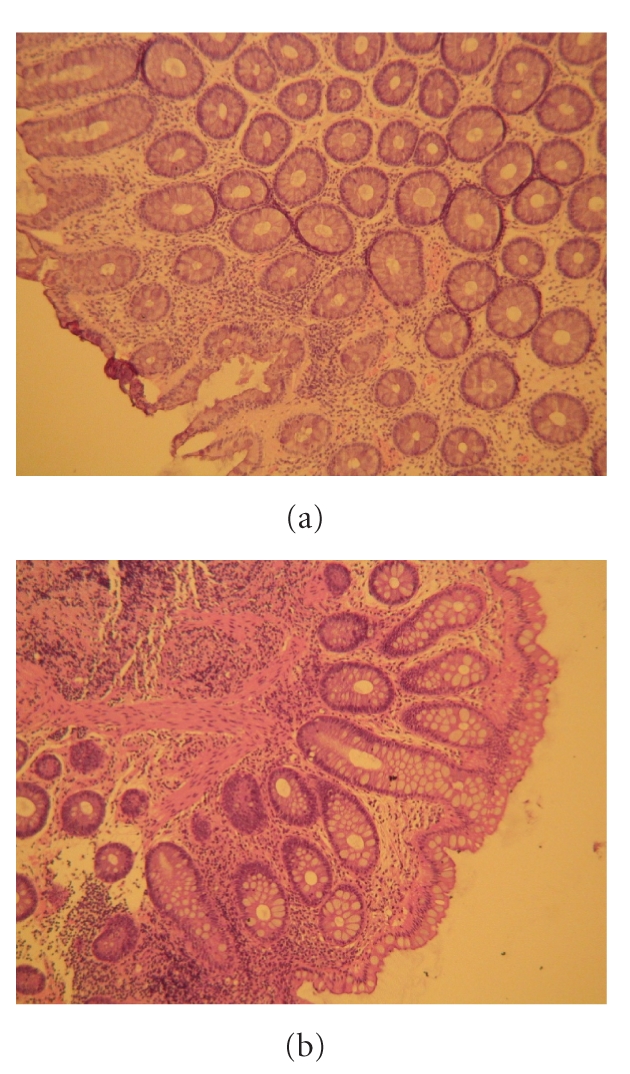
Histopathological evaluation of the large intestine at baseline (a) and at the onset of symptoms (b) revealed normal large intestine histology and mild chronic nonspecific colitis in the first patient with diarrhea, respectively.

**Table 1:** Comparison of epidemiological and large intestine histopathology data between untreated CHC patients and controls.

	CHC patients *n* = 24	Controls *n* = 10	*P* value
Gender (male/female)	14/10	6/4	1
Age (years)	37.04 ± 14.02	46.00 ± 13.38	.098
BMI (kg/m^2^)	23.09 ± 3.31	23.26 ± 3.78	.903
Inflammation (%)	14/24 (58.3%)	6/10 (60%)	1
Architectural disorder and decrease of mucus production (%)	6/24 (25%)	0/10 (0%)	.148
Paneth cells (%)	4/24 (16.7%)	0/10 (0%)	.296
Lymphoid follicles	1.33 ± 1.50	1.60 ± 1.43	.440

**Table 2:** Comparison of large intestine histopathology and diarrhea data of CHC patients before and after 24 *weeks* of treatment.

	CHC patients before treatment *n* = 15	CHC patients after 24 weeks of treatment *n* = 15	*P* value
Inflammation (%)	10/15 (66.7%)	8/15 (53.3%)	.71
Architectural disorder and decrease of mucus production (%)	6/15 (40%)	4/15 (26.7%)	.70
Paneth cells	4/15 (26.7%)	0/15	.10
Lymphoid follicles	1.33 ± 1.54	1.73 ± 1.62	.97
Diarrhea (%)	0/15	3/15 (20%)	.22
